# Decline in alcohol use among the general Czech population – role of young adults?

**DOI:** 10.1192/j.eurpsy.2025.357

**Published:** 2025-08-26

**Authors:** L. Kážmér, L. Csémy

**Affiliations:** 1 National Institute of Mental Health, Klecany, Czech Republic

## Abstract

**Introduction:**

Alcohol consumption, monitoring its prevalence and temporal trends, is one of the leading epidemiological interests in public health. When there are significant changes over time, understanding these new trends is crucial for health professionals. In the Czech Republic, a significant and sustained decline in alcohol use among the adolescent population has been observed since the mid-2010s (Kážmér & Orlíková *Adiktologie.* 2017; 17(2):118–132; Kážmér & Csémy *J Public Health Res.* 2019; 8(1):1493). The decline was in line with general trends across Europe and North America (Kraus *et al*. *Addiction.* 2018; 113(7):1317–1332). However, the factors driving the decline were attributed to new modes of adolescent leisure activities (Chomynová & Kážmér *J Subst Use.* 2019; 24(6):630–637) rather than changes in prevention-related issues (Pennay *et al*. *Drug Alcohol Rev.* 2018; 37(S1):S115–S119). If the decline among the adolescent population has continued into later age groups, there should also be a significant decline in today’s Czech young adults.

**Objectives:**

The study examines temporal trends in alcohol use in the general population of the Czech Republic, with an age-specific focus on young adults under 25 years of age.

**Methods:**

Time series of nationally representative data on alcohol use were compiled from the National Survey on Tobacco and Alcohol Use in the Czech Republic. The large-sample representative survey, conducted annually by the National Institute of Public Health, provided high-quality data on alcohol use among the general Czech population aged ≥15 years in 9 cross-sectional time periods: 2012, 2014, 2016, 2018–2023. The subgroup of respondents aged 15–24 years was selected. Time trends for three main measures on alcohol use were considered: i) mean annual alcohol consumption (in litres per person per year); ii) prevalence of binge drinking (%); iii) prevalence of abstainers (%).

**Results:**

Among the Czech population aged 15–24, all the three measures on alcohol use have improved in recent time periods. In particular, the estimated mean annual alcohol consumption decreased significantly from 8.9 litres (95% CI: 6.5–11.2) of pure ethanol per person per year in 2012 to 5.1 litres (95% CI: 4.0–6.2) in 2023. The prevalence of last year abstainers increased accordingly: from 8.6% (95% CI: 5.4%–12.8%) in 2012 to 13.1% (95% CI: 8.6%–17.6%) in 2023. In a similar vein, there was also an improvement in the prevalence of binge drinking.

**Conclusions:**

There was a noticeable decrease in alcohol use among the Czech young adults aged 15–24 years in recent periods, especially after 2020. Further research will be conducted to examine this trend in more detail.

**Funding:**

The study was supported by the Operational Program Johannes Amos Comenius, project number CZ.02.01.01/00/22_008/0004583.

**Disclosure of Interest:**

None Declared

